# Low income countries have the highest percentages of open access publication: A systematic computational analysis of the biomedical literature

**DOI:** 10.1371/journal.pone.0220229

**Published:** 2019-07-29

**Authors:** Jonathan Iyandemye, Marshall P. Thomas

**Affiliations:** University of Global Health Equity, Kigali, Rwanda; KU Leuven, BELGIUM

## Abstract

Open access publication rates have been steadily increasing over time. In spite of this growth, academics in low income settings struggle to gain access to the full canon of research literature. While the vast majority of open access repositories and funding organizations with open access policies are based in high income countries, the geographic patterns of open access *publication itself* are not well characterized. In this study, we developed a computational approach to better understand the topical and geographical landscape of open access publications in the biomedical research literature. Surprisingly, we found a strong negative correlation between country per capita income and the percentage of open access publication. Open access publication rates were particularly high in sub-Saharan Africa, but vastly lower in the Middle East and North Africa, South Asia, and East Asia and the Pacific. These effects persisted when considering papers only bearing authors from within each region and income group. However, papers resulting from international collaborations did have a higher percentage of OA than single-country papers, and inter-regional collaboration increased OA publication for all world regions. There was no clear relationship between the number of open access *policies* in a region and the percentage of open access *publications* in that region. To understand the distribution of open access across topics of biomedical research, we examined keywords that were most enriched and depleted in open access papers. Keywords related to genomics, computational biology, animal models, and infectious disease were enriched in open access publications, while keywords related to the environment, nursing, and surgery were depleted in open access publications. This work identifies geographic regions and fields of research that could be priority areas for open access advocacy. The finding that open access publication rates are highest in sub-Saharan Africa and low income countries suggests that factors other than open access policy strongly influence authors’ decisions to make their work openly accessible. The high proportion of OA resulting from international collaborations indicates yet another benefit of collaborative research. Certain applied fields of medical research, notably nursing, surgery, and environmental fields, appear to have a greater proportion of fee-for-access publications, which presumably creates barriers that prevent researchers and practitioners in low income settings from accessing the literature in those fields.

## Introduction

Open access (OA) describes materials that are free to access and read online, either through publisher websites or through publication repositories. It seems self-evident that OA publication maximizes the benefits of scientific findings for researchers, funders, and the public [1]. Some OA advocates now argue that all research publications should be openly accessible by default [2] and that access to knowledge stemming from research should be considered a fundamental human right [3]. In keeping with this, many government agencies, private foundations, and universities have concluded that the results of research they support should be openly accessible and have adopted mandates and policies to support OA publication. This has been accompanied by steady growth in OA repositories [4]. The most common routes to OA publication are either “gold” open access, which refers to papers that are made immediately available from the publisher under a creative commons license, or “green” OA papers, which are deposited by authors or publishers in a public repository. In addition, a large fraction of the literature is also made available on publisher’s websites without an explicit OA license. Most funders that have OA policies mandate that authors deposit papers in repositories, thus promoting the green OA publication route, but some have more recently established policies intended to promote gold OA [5]. Although there is evidence that OA policies and compliance efforts have increased OA publication [6], OA policies that promote green OA can place the burden of compliance upon authors, who may misunderstand OA or the policies.

In spite of growth in OA publication over time, more than 50% of newly-published research still can only legally be accessed with an institutional license or by paying publishers’ fees [7]. These fees are often too high for institutions and individuals based in low income countries [8,9], which has spurred initiatives to provide greater access to research literature in developing countries. The Research4Life initiative, a public-private partnership involving five United Nations agencies, provides free access to a large number of paywalled journals and books for organizations based in low income countries, notably through the HINARI program that is focused on biomedical research literature [9]. At the same time, services such as Sci-Hub have sprung up to provide access to pirated articles, thus circumventing publishers entirely. A recent study indicated that Sci-Hub provides access to a greater proportion of the published literature than a major US-based research university library [10]. This highlights the reality that the costs and complexities involved with licensing copyrighted research articles make adequate access a challenge, even for well-resourced universities in high income countries.

The “green” route of OA has been encouraged by funder policies, but also by an enormous growth in the number of OA repositories, particularly in Europe and North America. The number of OA repositories based in Africa lags far behind other parts of the world. According to the registry of open access repositories (ROAR), less than 4% of the total number of such repositories worldwide in 2018 were based in Africa [11]. Similarly, the vast majority of funding organizations with OA policies as of 2018 were based in Europe and North America, with less than 3% of total OA policies originating from organizations based in Africa [12]. It is generally believed that open access tracks with development [13] and that the Western world leads the OA movement due to technology and a more supportive publishing environment [14]. It has been speculated that publication fees, which are more common for OA papers, could have an inhibitory effect on OA publication by authors from low income countries [8,15], though such an inhibitory effect might be offset by fee waivers frequently granted to authors from these countries. Based on this confluence of factors, the prediction that OA publication rates are lower in low income countries than other countries seems very reasonable.

There is an increasing amount of evidence for a variety of benefits of OA publication, including economic, social, and academic benefits [15]. In academic circles, article citations are generally conflated with article importance, so one proxy for the impact of OA itself is the number of citations of OA articles compared to citations of similar non-OA articles. A majority of studies have identified a citation advantage for OA publications [14], and the OA citation advantage is true of international collaborations that focus on poverty-related diseases [16], though this advantage does vary by field of research and remains controversial [7]. OA articles are more likely to be accessed and downloaded than non-OA articles [17]. Some have speculated that OA publication will improve the consumption of scientific literature, but not the production of scientific research in developing countries [18], however, there is some evidence that providing free access to journal articles increases publication output of researchers based in low income countries [19]. The interaction between OA publication and international collaboration has not been studied extensively, but it is well established that the percentage of publications stemming from international collaborations is steadily increasing over time [20]. The research output of low income countries, particularly countries in sub-Saharan Africa, is also increasing [21]. In some countries in sub-Saharan Africa, papers resulting from international collaborations account for a large proportion of total research output [22], so it is important to account for the effects of international collaboration on the research output of these countries. It is well established that international collaborations tend to have a positive impact on the number of citations of papers [21,23,24].

The biomedical sciences have a higher percentage of OA publication than other fields of inquiry [7,25], presumably due to funders’ mandates and the utilitarian value of providing free access to biomedical research findings [18], but aside from research focused on specific diseases [16], we are not aware of studies that have evaluated OA across all different fields of study *within* the biomedical sciences. It seems likely that OA publication rates are not uniformly distributed across fields within biology and medicine, but the nature of this distribution is not known. Similarly, although the geographic distributions of OA *repositories* and *policies* are well documented, it is less clear how OA *publication* itself is globally distributed. In this study, we set out to determine the geographic distribution and topical distribution of OA publication in biomedical research indexed in PubMed.

## Methods

We used PubMed to identify a set of papers that matched specified search criteria. MEDLINE indexes journals that cover a broad array of topics, so we limited our search to papers in the biological sciences and medicine using MeSH terms and MeSH headings. The exact search term was: (“2015/01/01”[Date—Publication]: “2015/12/31"[Date—Publication]) AND (((Health Care category [mh]) OR (Psychiatry and Psychology category [mh])) OR ("Education"[MeSH Term]) OR ("Biological Science Disciplines"[MeSH Term])). These search criteria were designed to return a large body of literature, but restrict results to work in the biomedical sciences or medical education and exclude work in related fields, such as physics, mathematics, and the humanities (all of which also have MeSH terms). MeSH headings are hierarchical, and PubMed returns all papers that are below a given term in the hierarchy by default [26], so this search returned a very large volume of papers, comprising approximately 63% of all MEDLINE-indexed papers for 2015. We downloaded all of the PubMed IDs returned, then used the Entrez e-utilities to extract MeSH terms, digital object identifier (DOI), and affiliation metadata for each paper. We used Unpaywall [7] to identify the OA status of each paper, using the DOI to identify the paper. Unpaywall comprehensively tracks OA publications by compiling open access status from a wide variety of resources, institutional repositories, and databases. The affiliation strings were split into substrings using regular expressions, and we used a two-tiered approach to identify the country named in the substrings. We first identified countries of affiliation by their names, abbreviations, or major cities named in the affiliations. If this failed to yield a result, we submitted the affiliation substring to the google maps geocoding API [27]. For analysis of world economies and world regions, we used World Bank data from 2015 [28]. For analysis of enriched and depleted MeSH terms, we split terms into individual words, and tabulated all instances of each word. Word frequencies were normalized to total word counts, and words that were more than 33.33% enriched or depleted in OA papers relative to non-OA papers were considered. Words that appeared more than 4,000 times in the full set of words extracted from MeSH terms were considered for this portion of the analysis. All of the data extraction and processing was done with Python and the code is openly accessible on Github at https://github.com/iyandemye/oa_project.

## Results

To better understand the geographic and topical trends of OA publishing in biomedical research, we developed a computational pipeline that identifies authors’ country of affiliation from the text of affiliations provided by PubMed. We used PubMed to identify MEDLINE-indexed papers focused on biomedical sciences or medicine published in 2015. We ran the analysis on a small random sample (250 papers) of the full dataset and manually checked affiliations. In this sample, the method had a sensitivity of 99.7% and a false discovery rate of 0.5%. In the full dataset, of 643,138 papers matching the search criteria, 579,853 (90.2%) listed a text affiliation, and we identified at least one affiliation in 571,033 of these papers (98.5%). We identified OA status for 583,937 of the papers (90.8%). In total, we were able to establish OA status and at least one country of affiliation for 547,404 papers (85.1% of the full set of papers).

**Fig 1:**
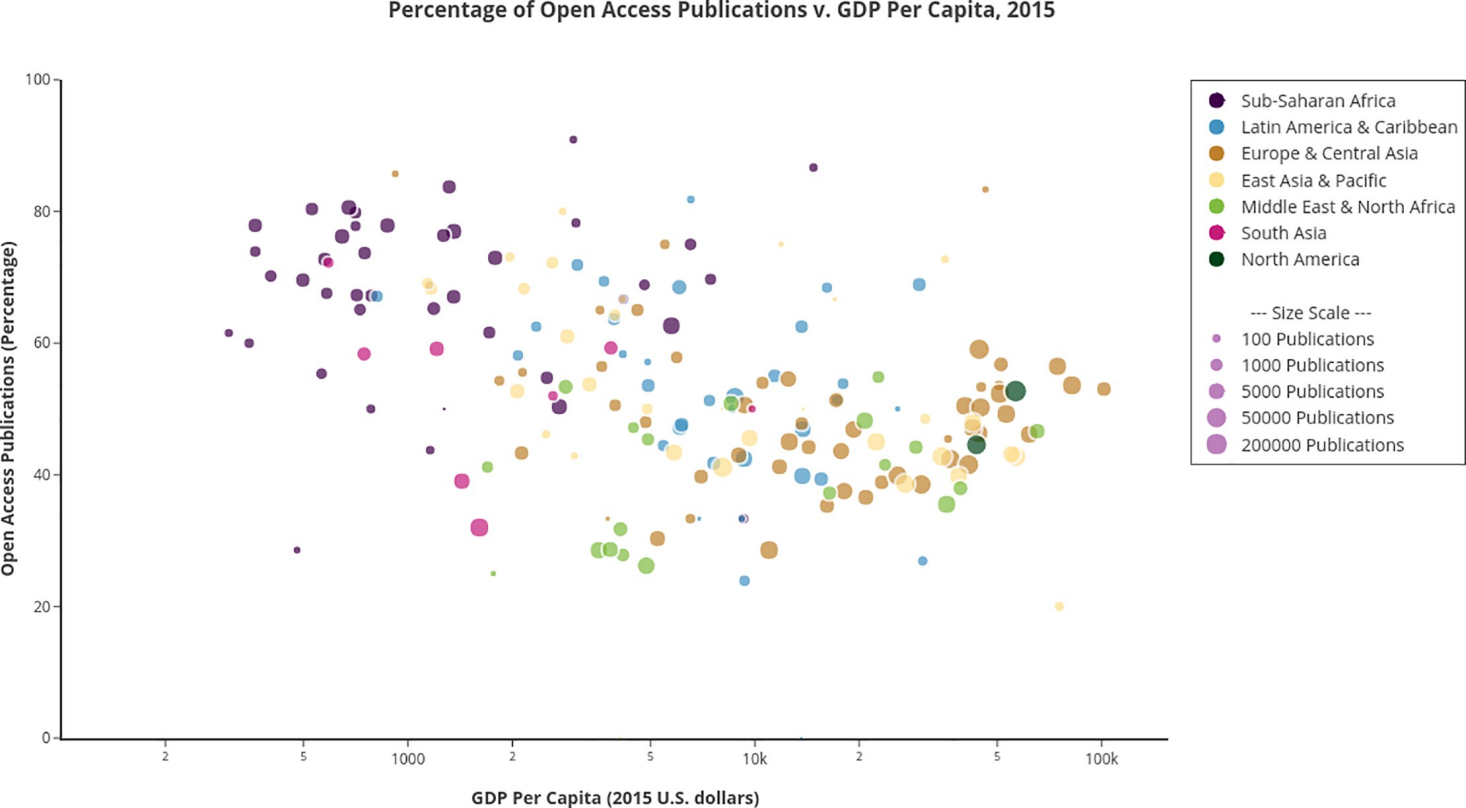
Geographic distribution of OA publications in biomedical research. Unique countries of affiliation (country-affiliations) were tabulated for each paper. For each country, the percentage of OA publication out of all papers with an affiliation in the country is reported. Per-capita income is from the World Bank. The size of each point is proportional to the log_2_-transformed number of publications.

We first evaluated the percentage of papers available in some open access form by country of authors’ affiliation. For these analyses, we counted unique countries of affiliation (country-affiliations) for each paper. In other words, a given paper with an author or authors affiliated in multiple countries is counted once in each of the unique countries represented amongst the affiliations on that paper. The total number of papers and percentage of OA for each country identified in the dataset is available in S1 Table. We compared the percentage of papers openly accessible by country, the number of papers published with authors affiliated in each country, and the per capita income [28] of each country (Fig 1). As expected, there were many more papers with authors based in high income countries [25], but surprisingly, the percentage of OA papers was generally higher in low income countries than in high income countries. There was a significant negative correlation between per capita GDP and the percentage of OA publications (r = -0.41, *P* = 1.8 x 10^−9^). We then performed the same analysis with countries binned by their World Bank income categories, focusing on all papers with affiliations in a given income group, as well as papers that only had authors within each income group. Interestingly, the percentage of OA publication was highest amongst papers with authors based in low income countries, lowest in papers with authors based in lower-middle and upper-middle income countries, and somewhat higher in papers with authors based in high income countries (Table 1). Although the trend persisted when accounting only for papers with author affiliations within a given income group, the percentage of OA papers dropped to varying degrees in all income groups. This suggests that collaborative research promotes OA, but collaboration alone cannot explain the high percentage of OA publication observed in the low income group or the low percentages in the middle income groups.

**Table 1:** Percentage of OA papers by country income brackets.

	*All* publications with authors in given group		Publications with authors only from given group	
World Bank Income Group	% OA	Number	% OA	Number
High Income	45.1	454484	45.0	403108
Upper Middle Income	42.1	102852	38.1	70138
Lower Middle Income	40.0	24627	30.6	13722
Low Income	72.9	4560	68.8	930

Authors’ countries of affiliation were binned by World Bank income groups. The percentage of OA is given for two subsets of the data: out of all papers published with any author affiliations in a given income group, and out of only those papers that have author affiliations within a given income group.

We also examined the distribution of OA publication by geographic region. The rates of OA publication were highest among papers with authors from sub-Saharan Africa and much lower in other geographic regions (Table 2). In single-region papers (those papers in which all affiliated authors were from a single geographic region), the trend was the same, suggesting that the regional effects observed are intrinsic to the publication incentives or preferences of investigators within regions. However, multi-regional papers (those papers with authors from two or more regions), had a higher percentage OA for every world region. We next examined the effect of international collaboration (regardless of region or income group) on OA publication. Across the entire dataset, we found that 41.0% of papers with a single country of affiliation were OA, whereas 57.2% of papers with multiple countries of affiliation were OA. Collectively, these results indicate that papers with authors based in sub-Saharan Africa, papers with authors based in low income countries, and papers resulting from international collaboration are all much more likely to be made openly accessible than papers that don’t have these properties.

**Table 2:** Percentage of OA papers by world region.

World Bank Region	% OA—all papers from region	% OA—single-region papers	% OA—multi-region papers	Number of OA Policies
Sub-Saharan Africa	66.3	55.3	72.5	29
North America	51.2	48.4	58.9	169
Latin America & Caribbean	49.3	46.8	53.5	54
Europe & Central Asia	43.5	39.1	57.8	596
East Asia & Pacific	41.3	36.9	55.2	88
Middle East & North Africa	34.9	26.4	46.7	5
South Asia	34.6	27.6	50.5	18

Authors’ countries of affiliation were binned by World Bank regions. The percentage OA by region is presented for three different groups of papers: papers with any affiliation in the region, papers with **only** that region in affiliations (single-region papers), and papers with that region **and at least one other region** of affiliation (multi-region papers). The number of OA policies tracked by ROARMAP [12] are also presented by region.

Interestingly, the pattern of OA *publication* does not match the pattern of OA *policies* instituted by funders, institutions, and governments. Sub-Saharan Africa has a low number of OA policies, but the highest percentage of OA publication of any region (Table 2). On the other hand, the two regions with the fewest OA policies—South Asia and the Middle East & North Africa—have the lowest percentage of OA publication. These results suggest a complex and non-linear relationship between OA policy and OA publication.

**Fig 2:**
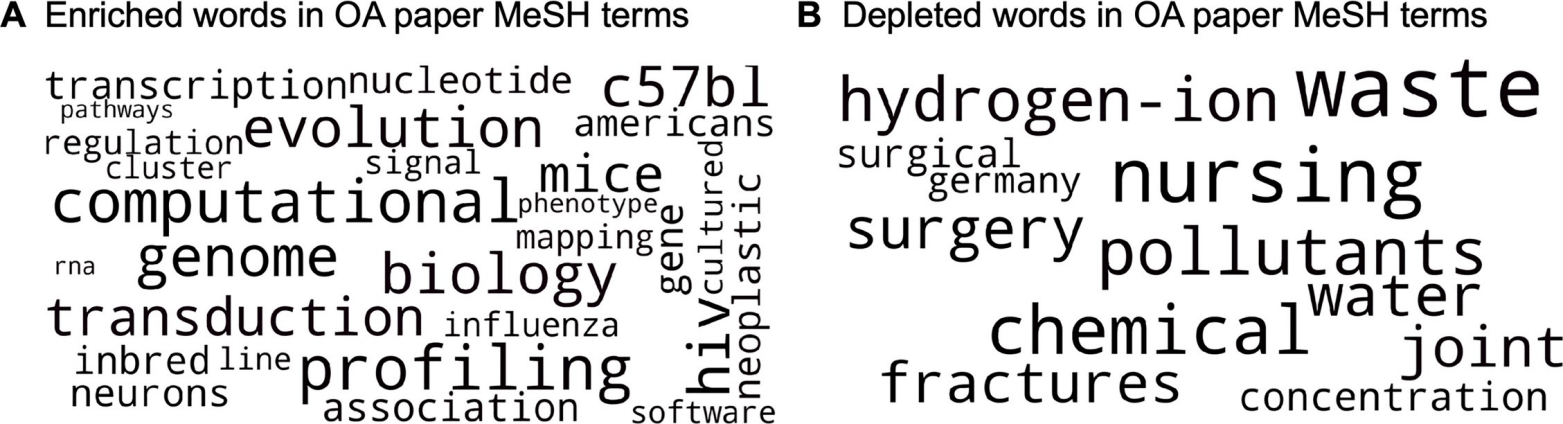
Enriched and depleted words in OA publications. MeSH terms were split into individual words and the total number of words were tabulated for OA and non-OA papers. Enrichment or depletion was computed from relative word frequency (the frequency of each word relative to all words within that group of papers). Words that were enriched or depleted by more than 33.3% in OA papers relative to all papers were considered for this analysis. (A) shows words that were more frequent in MeSH terms of OA papers (enriched words), while (B) shows words that were less frequent in MeSH terms of OA papers (depleted words). Enriched words were associated with computational biology, genomics, infectious diseases, and animal models, while depleted words were associated with surgery, nursing, and environmental issues.

We next examined whether there are differences in OA publication rates by field of study. PubMed does not classify papers by field *per se*, but all MEDLINE-indexed papers are manually tagged with MeSH terms [29], which provide an index of the topics covered in each publication. We analyzed the enrichment and depletion of individual words found in MeSH terms, comparing their frequency in OA publications vs. non-OA publications. A clear pattern emerges from this analysis. Terms associated with genomics, computational biology, infectious diseases, and mouse models were enriched in OA papers, while terms associated with environment, nursing, and surgery were depleted in OA papers (Fig 2). This suggests that certain topics of study are over-represented in OA publications, while applied topics in medicine, including research in nursing care and surgery, are under-represented in open-access publications. We found similar results when analyzing enrichment or depletion of full MeSH terms (S2 Table).

## Discussion

This work highlights unappreciated complexities in the geography of OA publication. The percentage of OA publication was highest in low income countries and particularly in sub-Saharan Africa, which has few OA policies and repositories, suggesting that factors in addition to OA policies play a major role in authors’ decisions to publish OA papers. We also observed a consistent effect of international and inter-regional collaboration: papers with authors based in multiple countries or regions had a substantially higher percentage of OA publication than their single-country and single-region counterparts.

We hypothesize that authors based in low income countries who routinely struggle to gain access to pay-to-view academic literature are motivated to make their work freely available to other researchers. However, there are other factors at play. First, research in low income countries may disproportionately come from fields that are over-represented in the OA literature and supported by funders with OA policies (such as research on infectious diseases like HIV and malaria). In support of this, other investigators have documented that research into poverty-related diseases has a very high percentage of open access publication [16], and in this study, MeSH terms related to these conditions are enriched in OA papers. Second, OA publication fee waivers offered to authors in low income countries are likely to encourage greater rates of OA publication by these authors. Finally, international collaboration certainly influences OA publication rates, though our own results indicate that collaboration alone cannot explain the high percentage of OA in sub-Saharan Africa and low income countries. It is likely that a combination of these factors influences authors’ decisions to publish OA papers. Subsequent studies could investigate the factors that impact OA publication decisions in low income countries.

The very low proportion of OA publication in certain areas—particularly the Middle East and North Africa and South Asia, points to geographic regions where additional work could be done to increase OA publication. Both of these regions have comparatively few institutions and funders with OA policies, but other factors may be involved in the low rates of OA publication. Notably, many OA journals only offer full fee waivers for low income countries; it is possible that partial fee waivers are insufficient to incentivize authors from middle income countries to submit their work to OA journals. The moderate percentage of OA publication in Europe and North America could be an indication of the success of OA policies that have been put in place by funders from these regions in recent years. Finally, the strong association between international collaboration and OA publication observed in this study indicates yet another benefit to collaborative research, in addition to other benefits that have been documented in previous work [21,23,24].

Our topical analysis also points to important trends in OA publication. It is concerning that keywords related to nursing, environmental health, and surgery are under-represented in the OA literature. This suggests that publications in these fields are more likely to require a fee for access than publications in other biomedical fields. Low income countries may have lax environmental regulations and high burdens of certain environmental contaminants [30], there is a large burden of untreated surgical disease in low income countries [31], and task-shifting increases the importance of nursing care in low income settings [32]. In other words, some of the fields of study that are *most applicable and actionable* in low income countries have a body of literature that is the *least accessible* to practitioners and researchers in these countries.

There are limitations to the current study. Not all journals report affiliations in PubMed, and affiliations are not formatted consistently between different journals. Moreover, by focusing on biomedical literature indexed in PubMed and tagged with MeSH terms, this work does not represent *all* published literature in 2015, rather it represents a *subset* of the literature that meets MEDLINE editorial standards [33,34]. To be returned by the search, papers had to be tagged with MeSH terms (therefore they had to be indexed by MEDLINE). This affords certain advantages: it allows for topical analysis of OA publication, and MEDLINE editorial standards substantially reduce the chance that so-called “predatory” journals will be indexed [35]. In addition to the relatively strict MEDLINE editorial criteria which undoubtedly bias PubMed results, it is well documented that different publication databases have variable representation of literature in different fields or from different countries [36]. Previous work has indicated that a large percentage of authors who publish in “predatory” journals are from Asia and Africa, so publications from these regions are likely to be under-represented in the current study [37]. In spite of these limitations, PubMed is a preferred resource for many biomedical researchers, and the results of this study are indicative of the accessibility of material returned by a PubMed search. Moreover, the results of this study are corroborated by another analysis, conducted using the Web of Science, in which the percentage OA was examined for selected countries in different world regions [38]. This suggests that the trends we observe in this paper may extend beyond the biomedical sciences and the PubMed database, but further research is needed to clarify this point. The Unpaywall database, which was used to determine OA status, is comprehensive, but there is no single definitive index of all OA publications, so it is also possible that some country or topic-level bias was introduced at the step of identifying OA status of each article. By working with publicly accessible data and making the computer code for these analyses freely available on Github, we hope to help others to replicate and build upon the work we have done here.

## Supporting information

S1 Table Total number of papers identified, and percentages OA, for all countries identified in the analysis. (CSV)

S2 Table Full MeSH term under- and over-representation in OA papers. Only terms with 500 or more counts in the full dataset were considered, and those that were 33.33% more or less enriched are presented here.
(CSV)
